# Socioeconomic and spatial distribution of depressive symptoms and access to treatment in Peru: A repeated nationwide cross-sectional study from 2014 to 2021

**DOI:** 10.1016/j.ssmph.2024.101724

**Published:** 2024-11-15

**Authors:** David Villarreal-Zegarra, Ali Al-kassab-Córdova, Sharlyn Otazú-Alfaro, Baltica Cabieses

**Affiliations:** a Instituto Peruano de Orientación Psicológica, Lima, Peru; b Department of Biomedical Informatics, School of Medicine, University of Utah, Salt Lake City, UT, United States; c Centro de Excelencia en Investigaciones Económicas y Sociales en Salud, Universidad San Ignacio de Loyola, Lima, Peru; d Centro de Salud Global Intercultural, Facultad de Medicina Clínica Alemana, Universidad del Desarrollo, Santiago, Chile; e Department of Health Sciences, University of York, York, United Kingdom

**Keywords:** Depression, Peru, PHQ-9, Inequality, Spatial analysis

## Abstract

**Background:**

Globally, evidence indicates that poverty and geographical setting influence the prevalence of depressive symptoms and access to treatment. Therefore, this study aimed to evaluate the socioeconomic and spatial distribution of depressive symptoms and treatment in Peru.

**Methods:**

We conducted an observational study based on the analysis of secondary data derived from the Peruvian Demographic and Health Surveys for 2014–2021. Using the Patient Health Questionnaire-9 on depressive symptoms, we estimated the Erreygers concentration index (ECI) to identify socioeconomic inequality in depressive symptoms and access to treatment. Spatial analyses were conducted using Global Moran's I, Kriging interpolation, hotspot analysis (Getis-Ord-Gi∗), and the Bernoulli-based Kulldorff spatial analysis.

**Results:**

The surveys included a total of 113,392 participants. Depressive symptoms exhibited only negative ECI values throughout the 2014–2021 period (pro-poor distribution), whereas access to treatment only displayed positive ECI values (pro-rich distribution). We identified two and four significant clusters in the southeastern areas of Peru in 2014 and 2021, respectively.

**Conclusions:**

Depressive symptoms were concentrated among the poorest, whereas access to treatment was remarkably concentrated among the wealthiest groups. A clustered spatial pattern was observed, and similar high-risk areas were identified. Social policies that address unequal socioeconomic and spatial distribution in depressive symptoms and treatment are required.

## Highlights

• Depressive symptoms were more concentrated among the poorest groups.
• Access to treatment had a greatly pro-rich distribution.
• The predicted prevalence of depressive symptoms was heterogeneous.
• High-risk clusters were identified in similar areas in both years under study.
• Social policies are required to tackle mental health disparities.

## 1 Introduction

Globally, depression has been the leading mental disorder in terms of disability-adjusted life-year burden with a 64% increase in prevalence between 1990 and 2019 (Global Burden of Disease [GBD] 2019 [Diseases and Injuries Collaborators], 2020; GBD, 2019 Mental Disorders Collaborators, 2022), while the economic burden of depression increases with age. The reason is that depression affects work productivity, while it impacts medical costs for the treatment of major depressive disorder (MDD) and its comorbidities as well as suicide-related costs (Greenberg et al., 2021; König, König, & Konnopka, 2019). For these reasons, depression is a major public health concern.

Access to treatment for depression varies according to the economic conditions (from 7% in low-income countries to 64% in high-income countries), being socioeconomic status (SES) a relevant factor in access to mental health services and receipt of health care for depression (Araya, Zitko, Markkula, Rai, & Jones, 2018; Mezzina, Gopikumar, Jenkins, Saraceno, & Sashidharan, 2022). The global mental health strategy for improving the mental health of populations focuses on the social determinants of mental health and on strengthening health services. At the level of health services, more than 75% of people with depressive symptoms remain untreated in low- and middle-income countries (Evans-Lacko et al., 2018). This finding implies inadequate and unequal access to treatment for depressive symptoms and MDD. For instance, in the United States, racial/ethnic inequality impacts access to treatment for depression, especially for racial minorities, such as African Americans and Hispanics, who receive treatment less frequently and are less likely to receive medication (McGregor et al., 2020). In Latin America, the circumstances are the same with racial/ethnic differences, region of residence, and economic income (related to employment, age, exposure to violence, and physical activity) as relevant determinants for the maintenance of this problem in Brazil (Mrejen, Hone, & Rocha, 2022). Conversely, at the level of social determinants, recognizing that not all people with depressive symptoms will perceive benefits from clinical treatments, such as psychotherapy or pharmacotherapy, is important. The reason is that these symptoms are typically linked to various conditions, such as poverty, social conflict, and underlying structural problems (Roberts et al., 2022). Two systematic reviews highlight that interventions that focus on improving social conditions, such as poverty reduction, exert a more positive impact on mental health than isolated clinical interventions (Elwadhi & Cohen, 2020; Finegan, Firth, Wojnarowski, & Delgadillo, 2018). These findings suggest that addressing the clinical aspects of depression alone is insufficient for achieving significant improvement without first changing the social context in which people live (Elwadhi & Cohen, 2020; Finegan et al., 2018).

Villarreal-Zegarra, Cabrera-Alva, Carrillo-Larco, and Bernabe-Ortiz (2020) conducted an observational study in Peru using trend analysis before the COVID-19 pandemic and reported no significant changes in the trend of the prevalence of depressive symptoms or access to treatment over time from 2014 to 2018. However, a time series analysis found that during the pandemic (since March 2020), the prevalence of moderate depressive symptoms increased and access to treatment for mild depressive symptoms improved. Conversely, there was an increase in moderate depressive symptoms without improvement in the access to treatment (Villarreal-Zegarra, Reátegui-Rivera et al., 2023). Existing socioeconomic inequalities may lead to poorer treatment access and outcomes for the less affluent, less educated, and unemployed (Antiporta et al., 2021; Hernández-Vásquez, Vargas-Fernández, Bendezu-Quispe, & Grendas, 2020).

This reality reinforces the need to promote public health policies that decrease inequality in access to treatment in Peru, which is related to socioeconomic gaps in the country. During the early months of the COVID-19 pandemic, the capacity of the Peruvian health system to address mental health (Villarreal-Zegarra, Segovia-Bacilio et al., 2023) and other non-COVID-19 health problems was significantly reduced (Villarreal-Zegarra, Bellido-Boza, Erazo, Pariona-Cárdenas, & Valdivia-Miranda, 2024). In addition, such socioeconomic gaps may be related to gender, age, level of education, area of residence, migrant status, and the spatial distribution of depressive symptoms. In particular, information regarding the spatial distribution of depressive symptoms in Peru is limited. Therefore, conducting an in-depth examination of spatial and socioeconomic distribution in relation to individuals with depressive symptoms, as well as access to treatment, is imperative. The current study intends to assess whether or not certain economic groups are more likely to exhibit depressive symptoms and whether or not disparities in access to treatment exists across socioeconomic strata. Prior to the pandemic, people from high-income brackets were found to be up to five times more likely to receive treatment than those from low-income brackets (Villarreal-Zegarra et al., 2020). This scenario underscores the need to evaluate this disparity in detail.

The current data at the peri-urban level revealed clusters of significantly high and low prevalence of cases with depressive symptomatology in the Lima Metropolitan area (Ruiz-Grosso et al., 2016). To the best of our knowledge, no study has evaluated the spatial distribution of depressive symptomatology in Peru at the national level and whether or not this spatial distribution has changed since the onset of the pandemic. The current evidence includes a stratified analysis of prevalence by region across Peru before the pandemic and reveals that the proportions of treated cases was highest and lowest in the coastal region (western zone) and southeastern and northeastern regions of Peru, respectively (Villarreal-Zegarra et al., 2020). However, this evidence was obtained only through a stratified analysis by region and not a detailed spatial analysis. Recognizing the importance of evaluating the socioeconomic and spatial distribution of depressive symptoms would promote improvement in the allocation of health resources for effective intervention and prevention. Consequently, the current study aimed to evaluate the socioeconomic distribution of depressive symptoms and their treatment and assess the spatial distribution of depressive symptoms throughout Peru. We also compared prepandemic and pandemic measures of COVID-19 to assess whether or not such an inequality has changed.

## 2 Material and methods

### 2.1 Study design and data source

This observational study derived data from the Demographic and Health Surveys (DHS) in Peru from 2014 to 2021. Since 2013, the Peruvian DHS has incorporated mental health questions into its evaluation. However, its initial subsample was relatively scant and not representative of the general population. From 2014 onward, data collected on mental health was representative at the national and departmental levels. Administratively, the Peruvian territory is divided into 24 departments, which are further subdivided into 196 provinces and 1845 districts. The National Institute of Statistics and Informatics (Spanish name: Instituto Nacional de Estadística e Informática [INEI]) conducts the Peruvian DHS annually through face-to-face interviews. During the COVID-19 pandemic in 2020, the data collection process was modified to be conducted via telephone. However, in 2021, the Peruvian DHS resumed face-to-face interviews.

### 2.2 Sampling and sample

The study employed two-stage probability sampling, which was representative of the national and regional levels of the Peruvian territory. At the first stage, clusters were selected as the primary sampling unit from information obtained from the latest census. While the DHS data is collected at the household level, the spatial data is collected at the sampling cluster level. Second, the secondary sampling unit (strata) was the Peruvian household. These data were obtained from cartographic reports and building registers (INEI, 2020). In addition, the sampling strategy was differentiated according to rural and urban areas. In rural areas, the primary units comprised groups of up to 2000 people; in urban areas, the units comprised blocks or block groups with more than 2000 persons and an average of 140 households. As such, the Peruvian DHS sample can be considered nationally and regionally representative. However, it is representative of men and women aged 15 years and older living in Peruvian households (in urban and rural areas) and excludes those who are incarcerated, hospitalized, or not living in the household under evaluation. Additional details regarding the sampling methodology are available in the Peruvian DHS technical documents (INEI, 2020).

### 2.3 Selection criteria

This study used data collected between 2014 and 2021 by the Peruvian DHS. The participants were men and women aged 15 years and older living in urban and rural areas across Peru with complete data on mental health assessment and the sociodemographic variables of interest.

### 2.4 Setting

Since 2012, Peru has been undergoing a mental health reform that has involved a shift from a model centered on large, highly complex hospitals to one focused on community mental health, improving access to mental health services for the population (Toyama et al., 2017). This reform resulted in the creation of the first 22 community mental health centers in 2015, a number that increased to 275 by January 2024. During the COVID-19 pandemic, the Peruvian government implemented various national policies and technical guidelines that were intended to improve the mental health of the general population, healthcare workers, people at risk of violence, and other specific groups. However, the implementation of these policies was partial, and health professionals and policymakers reported limitations in implementation (Mayo-Puchoc et al., 2023).

### 2.5 Variables

#### 2.5.1 Depressive symptoms

We defined depressive symptoms based on the nine indicators for MDD stated in the Diagnostic and Statistical Manual of Mental Disorders, Fifth Edition (DSM-5). The PHQ-9 was used to evaluate depressive symptoms over the past two weeks in the Peruvian population. This instrument was designed according to the DSM-IV criteria, which remain the same as those in the DSM-5. The PHQ-9 employs four-point Likert-type response options (0 = *not at all*; 1 = *several days*; 2 = *more than half of the days*; 3 = *almost every day*; Spitzer, Kroenke, & Williams, 1999) and presents valid and reliable evidence in the Peruvian context for people aged 15 years and older (Villarreal-Zegarra, Copez-Lonzoy, Bernabe-Ortiz, Melendez-Torres, & Bazo-Alvarez, 2019), and in students of 16–28 years in Peru (Huarcaya-Victoria et al., 2020). Meta-analyses on the PHQ-9 indicate that it possesses optimal levels of sensitivity and specificity when using the 10-or-higher-point cohort (Levis, Benedetti, & Thombs, 2019; Manea, Gilbody, & McMillan, 2015). Therefore, we dichotomized the variables into either with (≥10 points) or without (≤9 points) depressive symptoms (Levis et al., 2019; Manea et al., 2015). A cutoff of ≥10 points exhibits adequate levels of sensitivity (54.2; 95% CI 8.7–59.6) and specificity (87.4; 95% CI 85.2–89.3) in the Peruvian context (Villarreal-Zegarra, Barrera-Begazo et al., 2023). Notably, this study focused on the detection of depressive symptoms and not on the diagnosis of MDD, given that we used validated questionnaires instead of clinical interviews, which are conducted by health professionals.

#### 2.5.2 Access to treatment of depressive symptoms

The proportion of cases treated for depression was determined based on the number of participants with depressive symptoms who self-reported receiving treatment for depression by a health professional within the past 12 months. The variable was dichotomized according to whether or not they received treatment. Only participants with depressive symptoms were considered and not the entire population.

#### 2.5.3 Sociodemographic variables

We included the following sociodemographic variables: sex (male/female), age (15–34, 35–54, 55–74 and 75+ years), area of residence (urban/rural), and year of Peruvian DHS (2014–2021). These variables were used to characterize the study population and perform stratified inequality analysis.

#### 2.5.4 Socioeconomic status

The wealth index was employed as a proxy measurement of SES. It was calculated using principal components analysis based on information of household construction materials, access to sanitation, access to water, and ownership of various assets. Based on the information provided, the wealth index was created to classify individuals according to level of wealth (Rutstein & Johnson, 2004). However, asset-based approaches are valid for measuring inequality in living standards only in the absence of a direct measurement of SES (McKenzie, 2005). Previous studies, including some conducted in the Peruvian context, have still utilized the wealth index (Al-Kassab-Córdova, Silva-Perez, & Maguiña, 2022; Villarreal-Zegarra, Carrillo-Larco, & Bernabe-Ortiz, 2021).

### 2.6 Statistical analysis

We downloaded data on the Peruvian DHS from the “Microdatos” website of the INEI (http://iinei.inei.gob.pe/microdatos/). The INEI database was imported into STATA 16.0 (Stata Corporation, College Station, Texas, USA) for data description and inequality analysis. All analyses were performed using weights, strata, and clusters given the complex survey design. After filtering the data, they were exported to ArcGIS version 10.8 (ESRI, Redlands, CA, USA) and SaTScan™ for spatial analysis. Our study used different years of evaluations from the Peruvian DHS, each with its own specific complex sampling and weighting factors. Therefore, it is not possible to combine all the evaluation years without introducing bias. We combined all the databases and adjusted the weighting factors for each year individually. We then renormalised the pooled databases by constructing a single weighting factor for all years. All spatial data were projected to UTM Zone 18. Inequality analysis was performed yearly from 2014 to 2021, while spatial analysis was performed only for 2014 and 2021.

Inequality analysis was conducted using the *Lorenz* and *conindex* packages (Jann, 2016; O'Donnell, O'Neill, Van Ourti, & Walsh, 2016). We constructed concentration curves (CCs) of depressive symptoms and treatment using data from 2014 to 2021. CCs represent the cumulative proportion of depressive symptoms and treatment (y-axis) against wealth status (x-axis). A deviation in the curve to either side of the line of perfect equity (45-degree line) implies the presence of inequality. Deviations above and below the line indicate a pro-poor and pro-rich distribution, respectively. In other words, a pro-poor distribution indicates an event is more likely for poor people, whereas a pro-rich distribution suggests that an event is more likely for rich people. Moreover, to estimate the magnitude of inequality in both variables (having depressive symptoms and access to its treatment), we utilized the Erreygers concentration index (ECI). This is a normalized version of the traditional concentration index that overcomes certain boundaries related to the binary nature of outcome variables (depressive symptoms and their treatment) by satisfying certain properties such as transfer, level independence, cardinal invariance, and mirror. It is calculated as follows:

$$ECI={4\mu }_{h}*CI\text{,}$$

where $\mu_{h}$ represents the mean (i.e., proportion) of the variable under analysis, and CI represents the traditional concentration index (Erreygers, 2009). Subsequently, the ECI was computed for each sociodemographic variable. The Z-test was performed when comparing two groups assumed to have a large sample size. Meanwhile, the F-test was used when comparing more than two groups assumed to have equal variances (O'Donnell et al., 2016). In addition, we used 95% confidence intervals for the estimates whenever appropriate.

Regarding spatial analysis, we plotted the prevalence of both outcome variables in choropleth maps. Global Moran's I is a measure of spatial autocorrelation that assesses spatial distribution and ranges from −1 to +1. Accordingly, a positive value implies a clustered distribution, zero implies randomness, and a negative value implies a dispersed distribution. Hotspot analysis was performed using the Getis-Ord-Gi∗ statistic. To be considered a statistically significant hot spot, a feature should obtain a high *z*-score and a low *p*-value while being surrounded by other features with similar values (Getis & Ord, 1992). Red indicate significant hotspot areas (high clustering of depressive symptoms), while blue indicate significant cold spot areas (low clustering of depressive symptoms). Additionally, the study performed ordinary Kriging interpolation analysis to predict the prevalence of depressive symptoms in the unsampled areas based on the sampled areas. This step provided a continuous surface of the predicted prevalence in the entire study area.

The Kulldorff spatial analysis technique (Kulldorff, 1999), which is based on the Bernoulli model, employs a spatial scanning window that searches for high-risk clusters of depressive symptoms across the area under study. To fit the Bernoulli model, participants with and without depressive symptoms were considered as cases and controls, respectively. After ranking the clusters based on their LLR, the cluster with the highest LLR was considered the primary cluster, while the remaining clusters were considered secondary ones.

## 3 Results

### 3.1 Participant characteristics

A total of 113,392 participants were included from 2014 to 2021 out of which 10.1% (n = 27,633) and 13.1% (n = 32,436) belonged to the 2014 and 2021 Peruvian DHS, respectively. The majority of the participants in 2014 and 2021 were aged 15–34 years (43.1% and 42.9%, respectively), women (53.7% and 51.7%, respectively), and living in urban areas (75.1% and 80.7%, respectively). Furthermore, the majority achieved between 7 and 11 years of education (43.1% and 46.6%, respectively; Table 1). We performed a comparison between the characteristics of the participants and the population estimate for 2021 and found no significant differences between population characteristics (**Supplementary 1**).

**Table 1:** Sociodemographic characteristics of participants by year.

		2014		2021		Pooled 2014–2021	
		(n = 27,564)		(n = 32,436)		(n = 259,391)	
		n	%	n	%	n	%
Sex	Male	12,762	46.2%	13,863	48.3%	113,869	48.3%
	Female	14,802	53.8%	18,573	51.7%	145,522	51.7%
Ages (mean, SE)		40.6 (0.2)		40.8 (0.2)		40.6 (0.1)	

Age group	15–34	11,013	43.2%	15,881	42.9%	120,733	43.3%
	35–54	9370	33.9%	10,901	34.3%	86,784	34.3%
	55–74	5431	17.9%	4546	17.9%	40,930	17.4%
	75+	1750	5.0%	1108	5.0%	10,944	5.0%
Civil status	Married	16,276	57.8%	21,049	59.7%	169,019	61.3%
	Never married	6465	29.5%	5820	21.8%	49,837	23.4%
	Previously married	4823	12.7%	5567	18.5%	40,535	15.3%
Education level	Up to 6 years	10,142	29.7%	8171	21.8%	77,515	25.6%
	7–11 years	10,861	43.1%	15,035	46.6%	112,495	44.6%
	12+ years	6561	27.2%	9230	31.7%	69,381	29.8%
Area	Rural	10,636	25.0%	11,545	19.3%	91,323	24.0%
	Urban	16,928	75.1%	20,891	80.7%	168,068	76.0%
Regions	Metropolitan Lima	3255	31.4%	3850	35.3%	29,510	33.4%
	Rest of the Coast	7388	25.8%	9019	26.9%	73,815	25.5%
	Highlands	11,370	30.4%	11,573	24.7%	95,200	27.7%
	Jungle	5551	12.4%	7994	13.2%	60,866	13.3%
Wealth index	Very low	8129	18.7%	10,636	19.0%	79,416	20.6%
	Low	6767	19.2%	8138	20.4%	65,466	20.8%
	Middle	5140	19.9%	5874	20.8%	48,701	20.2%
	High	4091	21.0%	4563	20.0%	37,670	19.6%
	Very high	3437	21.2%	3225	19.8%	28,138	18.9%
Depressive symptoms	No	25,357	93.2%	30,350	93.1%	241,128	93.2%
	Yes	2207	6.8%	2086	7.0%	18,263	6.8%
Depressive cases treated in the last year ∗	No	2001	88.4%	1880	88.5%	16,573	89.3%
	Yes	2,06	11.6%	206	11.5%	1690	10.7%

Note: The two-stage sampling design was considered for estimations. ∗The number of participants includes only those with depressive symptoms.

### 3.2 Prevalence and treated cases of depressive symptoms

At the national level, the prevalence of cases with depressive symptoms in 2014 was 6.8% out of which 11.6% received treatment. The prevalence of depressive symptoms ranged from 4.0% to 14.7% among departments in 2014. In 2021, the national prevalence of cases with depressive symptoms was 6.9% out of which 11.5% received treatment. In this year, the prevalence of depressive symptoms ranged from 2.1% to 13.1% among departments (Fig. 1, Fig. 2A). When stratified according to the wealth index, the highest quintiles displayed more access to treatment for depressive symptoms in both years (Fig. 3).

**Fig. 1:**
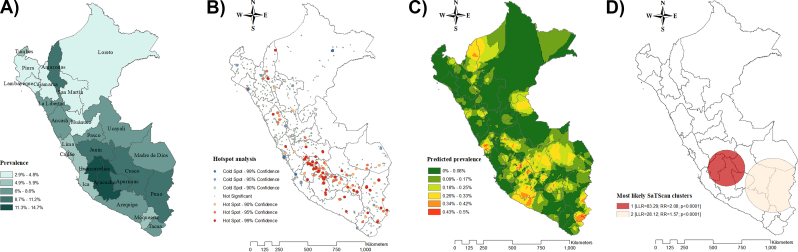
Spatial analysis of depressive symptoms in Peru in 2014. Fig. 1A. Choropleth map. Fig. 1B. Hotspot analysis (Getis-Ord-Gi∗). Fig. 1C. Kriging interpolation. Fig. 1D. Kulldorff's Bernoulli-based spatial scan statistics **Note:** Data were derived from the 2014 Peruvian Demographic and Health Survey. The choropleth map illustrates weighted proportions of depressive symptoms. In the hotspot analysis, red areas represent significant hotspots (regions with a high clustering of depressive symptoms), while blue areas indicate significant cold spots (regions with low clustering). Ordinary Kriging was used for spatial interpolation. The cluster analysis displays only statistically significant clusters. (For interpretation of the references to colour in this figure legend, the reader is referred to the Web version of this article.)

**Fig. 2:**
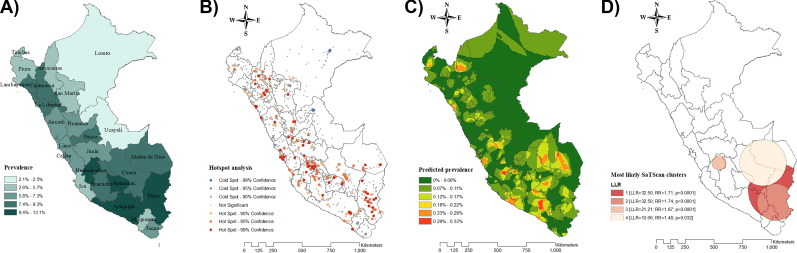
Spatial analysis of depressive symptoms in Peru in 2021. Fig. 2A. Choropleth map. Fig. 2B. Hotspot analysis (Getis-Ord-Gi∗). Fig. 2C. Kriging interpolation. Fig. 2D. Kulldorff's Bernoulli-based spatial scan statistics. **Note:** Data were derived from the 2021 Peruvian Demographic and Health Survey. The choropleth map illustrates weighted proportions of depressive symptoms. In the hotspot analysis, red areas represent significant hotspots (regions with a high clustering of depressive symptoms), while blue areas indicate significant cold spots (regions with low clustering). Ordinary Kriging was used for spatial interpolation. The cluster analysis displays only statistically significant clusters. (For interpretation of the references to colour in this figure legend, the reader is referred to the Web version of this article.)

**Fig. 3:**
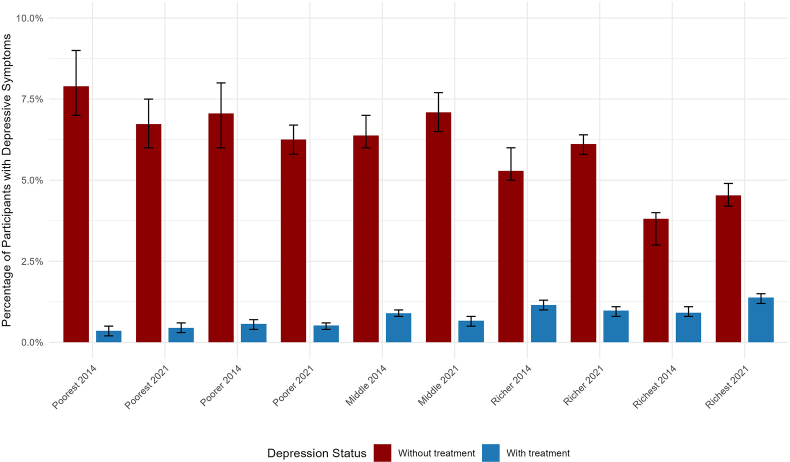
Treatment of depressive symptoms according to wealth index in 2014 and 2021 **Note:** Wighted proportions were reported.

### 3.3 Inequality analysis

**Fig. 4:**
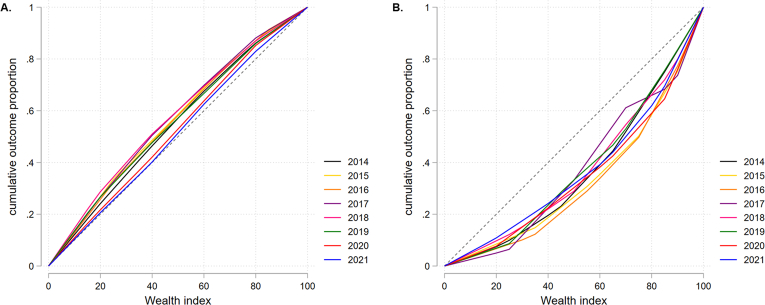
Inequality analysis from 2014 to 2021. Fig. 4A. Depressive symptoms. Fig. 4B. Treatment of depressive symptoms. **Note:** The plots display concentration curves (also known as Lorenz curves). The x-axis represents the wealth index, while the y-axis depicts the cumulative proportion of depressive symptoms and access to treatment (among participants with depressive symptoms). The diagonal line represents perfect equality. Deviations from this line indicate levels of inequality in the distribution of these outcomes.

Depressive symptoms obtained only negative ECI values in the general population for the 2014–2021 period, which implies that inequality was concentrated among the poorest populations (Fig. 4A). Contrarily, access to treatment for depressive symptoms only obtained positive ECI values, which reveals that it is concentrated among the wealthiest populations in the years under study (Fig. 4B). **Supplementary 2** summarizes the ECI values and confidence intervals and indicates that although the baseline estimates may vary across years, the different ECI measurements fall within very similar confidence intervals.

When stratifying the analysis by sociodemographic variables, the magnitude of inequality in depressive symptoms was greater in older participants than among younger individuals. Additionally, those previously married exhibited a higher magnitude of pro-poor inequality in depressive symptoms. Participants with 7–12 years of education exhibited lower inequality in depressive symptoms than those with up to 6 years or more than 12 years of education. This means that we did not find a social gradient in socioeconomic inequality of depressive symptoms by education level; yet participants with 7–12 years of education exhibited a higher magnitude of pro-rich inequality. Living in Lima Metropolitan area or the Highlands was also associated with a higher magnitude of inequality in depressive symptoms than did living in the rest of the Coast or the Jungle (see Table 2). Conversely, the magnitude of inequality in access to treatment was higher among the younger age groups and those who were never married. In addition, participants with 7–12 years of education exhibited a higher magnitude of pro-rich inequality. The magnitude of inequality (pro-rich) in access to treatment was higher among those living in urban areas and the Lima Metropolitan area. However, none of these findings were sustained in all years (see Table 3).

**Table 2:** Erreygers concentration index of depressive symptoms by sociodemographic characteristics and year.

	2014	2015	2016	2017	2018	2019	2020	2021
	ECI	ECI	ECI	ECI	ECI	ECI	ECI	ECI
**Overall**	−0.0268 (−0.017 to −0.0366)	−0.0396 (−0.0298 to −0.0494)	−0.0361 (−0.0261 to −0.0461)	−0.036 (−0.027 to −0.045)	−0.0362 (−0.0268 to −0.0456)	−0.0296 (−0.0206 to −0.0386)	−0.0128 (−0.0038 to −0.0218)	−0.0069 (0.0033 to −0.0171)

**Sex**								
Male	−0.0187 (−0.0067 to −0.0307)	−0.0373 (−0.0257 to −0.0489)	−0.0341 (−0.0237 to −0.0445)	**−0.0264 (-0.0164 to -0.0364)**	−0.0295 (−0.0183 to −0.0407)	−0.0351 (−0.0251 to −0.0451)	−0.006 (0.0044 to −0.0164)	−0.0103 (0.0026 to −0.0232)
Female	−0.0355 (−0.0208 to −0.0502)	−0.0433 (−0.0278 to −0.0588)	−0.0393 (−0.0232 to −0.0554)	**−0.0456 (-0.0319 to -0.0593)**	−0.0436 (−0.0299 to −0.0573)	−0.0256 (−0.0115 to −0.0397)	−0.0196 (−0.0057 to −0.0335)	−0.0041 (0.0104 to −0.0186)

**Age group**								
15–34	−0.0053 (0.0045 to −0.0151)	0.0006 (0.0098 to −0.0086)	**0.0119 (0.0229 to 0.0009)**	**0.0017 (0.0131 to -0.0097)**	**0.0009 (0.0107 to -0.0089)**	0.0021 (0.0131 to −0.0089)	0.0142 (0.0264–0.002)	**0.03 (0.0453 to 0.0147)**
35–54	−0.0261 (−0.01 to −0.0422)	−0.0297 (−0.0144 to −0.045)	**−0.0373 (-0.0222 to -0.0524)**	**−0.038 (-0.0241 to -0.0519)**	**−0.0421 (-0.028 to -0.0562)**	−0.0228 (−0.0089 to −0.0367)	−0.0171 (−0.004 to −0.0302)	**−0.0222 (-0.0087 to -0.0357)**
55–74	−0.0535 (−0.0239 to −0.0831)	−0.1222 (−0.0912 to −0.1532)	**−0.1019 (-0.0762 to -0.1276)**	**−0.1028 (-0.0799 to -0.1257)**	**−0.0943 (-0.0714 to -0.1172)**	−0.0813 (−0.056 to −0.1066)	−0.0489 (−0.0264 to −0.0714)	**−0.0332 (-0.006 to -0.0604)**
75+	−0.1243 (−0.0737 to −0.1749)	−0.1798 (−0.1173 to −0.2423)	**−0.1791 (-0.1164 to -0.2418)**	**−0.1588 (-0.1067 to -0.2109)**	**−0.1288 (-0.0586 to -0.199)**	−0.1669 (−0.1059 to −0.2279)	−0.1197 (−0.0617 to −0.1777)	**−0.1337 (-0.0755 to -0.1919)**

**Civil status**								
Married	**−0.025 (-0.0121 to -0.0379)**	−0.0328 (−0.0218 to −0.0438)	−0.0342 (−0.0226 to −0.0458)	**−0.0338 (-0.0244 to -0.0432)**	−0.034 (−0.0244 to −0.0436)	−0.0211 (−0.0099 to −0.0323)	**−0.0128 (-0.0028 to -0.0228)**	**−0.0116 (-0.0014 to -0.0218)**
Never married	**−0.0077 (0.0052 to -0.0206)**	−0.0124 (0.0029 to −0.0277)	0.0016 (0.0173 to −0.0141)	**0.0034 (0.022 to -0.0152)**	−0.0016 (0.0162 to −0.0194)	−0.0035 (0.0143 to −0.0213)	**0.0279 (0.0455 to 0.0103)**	**0.0316 (0.0565 to 0.0067)**
Previously married	**−0.0628 (-0.0316 to -0.094)**	−0.1106 (−0.0726 to −0.1486)	−0.0906 (−0.0563 to −0.1249)	**−0.0951 (-0.0649 to -0.1253)**	−0.0877 (−0.0569 to −0.1185)	−0.0951 (−0.0684 to −0.1218)	**−0.0692 (-0.0429 to -0.0955)**	**−0.0425 (-0.0155 to -0.0695)**

**Area**								
Rural	−0.0121 (0.003 to −0.0272)	−0.0266 (−0.0125 to −0.0407)	−0.022 (−0.0085 to −0.0355)	−0.0178 (−0.0064 to −0.0292)	−0.0289 (−0.0181 to −0.0397)	−0.0186 (−0.0066 to −0.0306)	−0.0141 (−0.0035 to −0.0247)	−0.0151 (−0.0051 to −0.0251)
Urban	−0.0259 (−0.0143 to −0.0375)	−0.0301 (−0.0189 to −0.0413)	−0.0295 (−0.0191 to −0.0399)	−0.0287 (−0.0187 to −0.0387)	−0.0248 (−0.014 to −0.0356)	−0.0168 (−0.0064 to −0.0272)	−0.0135 (−0.0031 to −0.0239)	−0.0128 (−0.0008 to −0.0248)

**Region**								
Metropolitan Lima	**−0.0225 (-0.0015 to -0.0435)**	**−0.0425 (-0.0186 to -0.0664)**	−0.031 (−0.011 to −0.051)	**−0.0281 (-0.0093 to -0.0469)**	**−0.0262 (-0.0068 to -0.0456)**	−0.0161 (0.0008 to −0.033)	**−0.0169 (0.0007 to -0.0345)**	**−0.0173 (0.0035 to -0.0381)**
Rest of the Coast	**−0.0121 (0.0022 to -0.0264)**	**−0.0066 (0.0087 to -0.0219)**	−0.0225 (−0.0047 to −0.0403)	**−0.0179 (-0.0052 to -0.0306)**	**−0.0064 (0.0087 to -0.0215)**	−0.0238 (−0.0075 to −0.0401)	**−0.0028 (0.0135 to -0.0191)**	**−0.0022 (0.0125 to -0.0169)**
Highlands	**−0.0231 (-0.007 to -0.0392)**	**−0.0443 (-0.028 to -0.0606)**	−0.0364 (−0.0199 to −0.0529)	**−0.0406 (-0.0251 to -0.0561)**	**−0.0593 (-0.0424 to -0.0762)**	−0.0419 (−0.0313 to −0.0525)	**−0.0026 (0.0097 to -0.0149)**	**0.0031 (0.0205 to -0.0143)**
Jungle	**−0.0153 (0.0021 to -0.0327)**	**−0.0004 (0.0149 to -0.0157)**	0.0091 (0.0242 to −0.006)	**0.0136 (0.0289 to -0.0017)**	**−0.0015 (0.0154 to -0.0184)**	−0.0013 (0.0095 to −0.0121)	**0.0143 (0.0292 to -0.0006)**	**0.0117 (0.0309 to -0.0075)**

**Education level**								
Up to 6 years	**0.0222 (0.0459 to -0.0015)**	**−0.025 (-0.0056 to -0.0444)**	**−0.0171 (0.0029 to -0.0371)**	**−0.0146 (0.0081 to -0.0373)**	−0.0106 (0.0145 to −0.0357)	**0.0057 (0.0235 to -0.0121)**	**0.0079 (0.0281 to -0.0123)**	**0.0092 (0.0327 to -0.0143)**
7–11 years	**0.0037 (0.0178 to -0.0104)**	**0.0021 (0.0137 to -0.0095)**	**0.0171 (0.0312 to 0.003)**	**0.0126 (0.0281 to -0.0029)**	0.0132 (0.0277 to −0.0013)	**0.0152 (0.0297 to 0.0007)**	**0.1226 (0.1369 to 0.1083)**	**0.0245 (0.0392 to 0.0098)**
12+ years	**−0.0051 (0.0102 to -0.0204)**	**−0.0189 (-0.004 to -0.0338)**	**−0.0211 (-0.0082 to -0.034)**	**−0.0255 (-0.0149 to -0.0361)**	−0.0159 (−0.0053 to −0.0265)	**−0.0167 (-0.0061 to -0.0273)**	**−0.0171 (-0.0046 to -0.0296)**	**−0.0127 (0.002 to -0.0274)**

Note: The two-stage sampling design was considered for estimations. ECI: Erreygers concentration index. Values in blond were significative (*p*-Value <0.05; test for statistically significant differences with Ho: diff = 0). Z-test was performed when comparing two groups in which a large sample size was assumed. F-test was used when comparing more than two groups in which equal variances were assumed.

**Table 3:** Erreygers concentration index of treatment access for depressive symptoms by sociodemographic characteristics and year.

	2014	2015	2016	2017	2018	2019	2020	2021
	ECI	ECI	ECI	ECI	ECI	ECI	ECI	ECI
**Overall**	0.1288 (0.1852–0.0724)	0.1293 (0.1722–0.0864)	0.1375 (0.1794–0.0956)	0.0963 (0.1531–0.0395)	0.1062 (0.157–0.0554)	0.1243 (0.178–0.0706)	0.1378 (0.1974–0.0782)	0.1204 (0.1765–0.0643)

**Sex**								
Male	0.1303 (0.2393–0.0213)	0.1061 (0.1786–0.0336)	0.0952 (0.1736–0.0168)	0.0696 (0.1407 to −0.0015)	0.0904 (0.1647–0.0161)	**0.0441 (0.1129 to -0.0247)**	0.1122 (0.2063–0.0181)	0.1044 (0.2261 to −0.0173)
Female	0.1273 (0.1937–0.0609)	0.1395 (0.1895–0.0895)	0.1498 (0.1998–0.0998)	0.105 (0.1728–0.0372)	0.1101 (0.1722–0.048)	**0.1482 (0.2143 to 0.0821)**	0.151 (0.2255–0.0765)	0.1251 (0.1847–0.0655)

**Age group**								
15–34	**0.1747 (0.2937 to 0.0557)**	0.1147 (0.2364 to −0.007)	**0.1378 (0.195 to 0.0806)**	0.0636 (0.1675 to −0.0403)	**0.1243 (0.2282 to 0.0204)**	**0.0588 (0.1497 to -0.0321)**	0.1237 (0.197–0.0504)	**0.1171 (0.2088 to 0.0254)**
35–54	**0.1913 (0.313 to 0.0696)**	0.1231 (0.1915–0.0547)	**0.1338 (0.2098 to 0.0578)**	0.0998 (0.1874–0.0122)	**0.103 (0.1939 to 0.0121)**	**0.1217 (0.2013 to 0.0421)**	0.1361 (0.2165–0.0557)	**0.1439 (0.2394 to 0.0484)**
55–74	**0.0201 (0.0773 to -0.0371)**	0.162 (0.238–0.086)	**0.1135 (0.1895 to 0.0375)**	0.0735 (0.1468–0.0002)	**0.109 (0.1886 to 0.0294)**	**0.1715 (0.267 to 0.076)**	0.1388 (0.2327–0.0449)	**0.1169 (0.1982 to 0.0356)**
75+	**0.1079 (0.2118 to 0.004)**	0.0599 (0.1538 to −0.034)	**0.112 (0.1853 to 0.0387)**	0.1665 (0.2604–0.0726)	**0.0596 (0.1564 to -0.0372)**	**0.0763 (0.1731 to -0.0205)**	0.1163 (0.2223–0.0103)	**0.0162 (0.1399 to -0.1075)**

**Civil status**								
Married	0.1087 (0.191–0.0264)	0.1235 (0.2632 to −0.0162)	**0.1077 (0.2059 to 0.0095)**	0.0808 (0.257 to −0.0954)	**0.0676 (0.2422 to -0.107)**	0.1561 (0.3162 to −0.004)	**0.1507 (0.2818 to 0.0196)**	**0.0764 (0.1462 to 0.0066)**
Never married	0.1786 (0.2987–0.0585)	0.1188 (0.189–0.0486)	**0.233 (0.3071 to 0.1589)**	0.0531 (0.1335 to −0.0273)	**0.1624 (0.2579 to 0.0669)**	0.0428 (0.1241 to −0.0385)	**0.1485 (0.244 to 0.053)**	**0.1811 (0.2871 to 0.0751)**
Previously married	0.1305 (0.2287–0.0323)	0.1338 (0.2079–0.0597)	**0.1363 (0.2143 to 0.0583)**	0.1192 (0.2041–0.0343)	**0.1363 (0.2318 to 0.0408)**	0.1084 (0.1933–0.0235)	**0.0549 (0.1488 to -0.039)**	**0.1162 (0.1975 to 0.0349)**

**Area**								
Rural	0.0268 (0.066 to −0.0124)	**0.0345 (0.0715 to -0.0025)**	0.0471 (0.0822–0.012)	0.0182 (0.0539 to −0.0175)	0.0578 (0.1046–0.011)	0.015 (0.052 to −0.022)	**0.0166 (0.0834 to -0.0502)**	**0.0302 (0.1149 to -0.0545)**
Urban	0.1082 (0.1797–0.0367)	**0.1274 (0.1966 to 0.0582)**	0.1173 (0.1786–0.056)	0.0388 (0.1084 to −0.0308)	0.1054 (0.1677–0.0431)	0.082 (0.16–0.004)	**0.1302 (0.2141 to 0.0463)**	**0.1245 (0.2035 to 0.0455)**

**Region**								
Metropolitan Lima	**0.1351 (0.2794 to -0.0092)**	**0.1028 (0.2759 to -0.0703)**	0.1098 (0.2409 to −0.0213)	0.0968 (0.2552 to −0.0616)	0.1227 (0.2379–0.0075)	0.0491 (0.14 to −0.0418)	0.1079 (0.2083–0.0075)	**0.1026 (0.2302 to -0.025)**
Rest of the Coast	**0.0854 (0.1732 to -0.0024)**	**0.1822 (0.272 to 0.0924)**	0.099 (0.1707–0.0273)	0.0481 (0.1183 to −0.0221)	0.0168 (0.1077 to −0.0741)	0.0671 (0.158 to −0.0238)	0.1132 (0.2136–0.0128)	**0.061 (0.1327 to -0.0107)**
Highlands	**0.0224 (0.0591 to -0.0143)**	**0.091 (0.1398 to 0.0422)**	0.1045 (0.1461–0.0629)	0.0934 (0.1469–0.0399)	0.1093 (0.1656–0.053)	0.0882 (0.167–0.0094)	0.1022 (0.1922–0.0122)	**0.0968 (0.1756 to 0.018)**
Jungle	**0.1469 (0.2467 to 0.0471)**	**0.0931 (0.1574 to 0.0288)**	0.0956 (0.1372–0.054)	0.0946 (0.1565–0.0327)	0.0861 (0.1618–0.0104)	0.0663 (0.1451 to −0.0125)	0.1044 (0.1944–0.0144)	**0.0463 (0.1359 to -0.0433)**

**Education level**								
Up to 6 years	**0.0695 (0.1297 to 0.0093)**	**0.0721 (0.1211 to 0.0231)**	**0.0385 (0.0669 to 0.0101)**	**0.0711 (0.1152 to 0.027)**	0.0969 (0.1684–0.0254)	**0.0704 (0.1631 to -0.0223)**	**0.0393 (0.1397 to -0.0611)**	**0.0221 (0.0833 to -0.0391)**
7–11 years	**0.1668 (0.2685 to 0.0651)**	**0.0551 (0.1359 to -0.0257)**	**0.1297 (0.2134 to 0.046)**	**0.1058 (0.222 to -0.0104)**	0.0831 (0.1758 to −0.0096)	**0.1226 (0.2271 to 0.0181)**	**0.1784 (0.2788 to 0.078)**	**0.0849 (0.1933 to -0.0235)**
12+ years	**0.1342 (0.2843 to -0.0159)**	**0.1848 (0.3012 to 0.0684)**	**0.0961 (0.2264 to -0.0342)**	**−0.0644 (0.0575 to -0.1863)**	0.1322 (0.2414–0.023)	**−0.0012 (0.1317 to -0.1341)**	**0.0804 (0.1808 to -0.02)**	**0.1777 (0.3045 to 0.0509)**

**Note:** The two-stage sampling design was considered for estimations. ECI: Erreygers concentration index. The number of participants includes only those with depressive symptoms. Values in blond were significative (*p*-Value <0.05; test for statistically significant differences with Ho: diff = 0). Z-test was performed when comparing two groups in which a large sample size was assumed. F-test was used when comparing more than two groups in which equal variances were assumed.

### 3.4 Spatial analysis

A clustered spatial distribution pattern of depressive symptoms was found in 2014 and 2021 (Global Moran's I = 0.007 and 0.021, respectively; **Supplementary 3**). Hotspot analysis (Getis-Ord-Gi∗ statistic) identified areas with high-intensity clustering of depressive symptoms, which were found in the central and south regions in 2014, and in the northwest, central, and southeast regions in 2021 (Fig. 1, Fig. 2B). The Kriging interpolation predicted that in 2014, the expected prevalence of depressive symptoms was higher in the southern departments of Peru such as Arequipa, Ayacucho, Apurímac, Cusco, and Madre de Dios. In 2021, the predicted prevalence of depressive symptoms remained high in southern Peru, particularly in the departments of Arequipa, Madre de Dios, and Cusco (Fig. 1, Fig. 2C).

The Bernoulli-based spatial scan statistics identified 414 and 741 high-risk clusters of depressive symptoms in 2014 and 2021 out of which 2 and 4 were statistically significant, respectively. In both scenarios, the primary cluster was located in the southeast, while the secondary clusters were observed in the surrounding areas (Fig. 1, Fig. 2D).

## 4 Discussion

### 4.1 Main findings

Socioeconomic inequalities in depressive symptoms and access to treatment were steadily distributed among the poor and rich, respectively. We also observed that greater inequality existed in access to treatment compared with the presence of depressive symptoms. In other words, patients do not have access to certain forms of intervention to address depressive symptoms, which should be comprehensive and delivered by physicians, psychologists, nurses, social workers, and other health professionals as appropriate. However, the magnitude of the inequality in depressive symptoms, which was pro-poor, slightly decreased in 2020 and 2021. Those living in rural areas or the Lima Metropolitan area exhibited a higher magnitude of pro-rich inequality in access to treatment. This finding underscored the necessity to conduct an in-depth analysis to understand the spatial patterns. In fact, the spatial distribution of depressive symptoms revealed clustering with similar findings for 2014 and 2021. In addition, the predicted prevalence of depressive symptoms was heterogeneous within and between departments, and high-risk areas in southeastern departments were identified. In line with these results, the study noted a similar prevalence of depression among the years under study. These stagnant patterns may be due to insufficient government effort. Moreover, the similar clustered spatial pattern identified in 2014 and 2021 pointed to the presence of potential underlying factors among departments. A few of the departments embedded in these clusters are Cusco, Puno, Madre de Dios, Huancavelica, and Ayacucho, which obtained the lowest human development index values at the national level.

Inequality in access to treatment, measured using the wealth index as the asset index, increases with higher levels of education. High levels of education are associated with high-income levels, exacerbating disparities in access to resources between the rich and the poor. This phenomenon is particularly evident in the Lima Metropolitan area, where inequality is greater than in other regions. The Lima Metropolitan area is host to the largest number of public health centers in Peru, but it is also home to the largest proportion of the Peruvian population, which could lead to long wait times, difficulty in setting appointments, and the need to sacrifice working hours to wait for treatment in the public system. These inconveniences could exacerbate inequalities compared with private services in which access is facilitated by payment for care. However, poor people cannot afford this type of private treatment.

### 4.2 Comparison with other studies

Based on the analysis, approximately 1 out of 20 Peruvians suffer from depressive symptoms, and they were concentrated among the poorest groups, although at a low magnitude. This finding agrees with those of other studies from the United States (Korous et al., 2022) and European countries (Freeman et al., 2016; Hoebel, Maske, Zeeb, & Lampert, 2017). In Peru, individuals belonging to the highest wealth index quintile were associated with 50% lower odds of presenting depressive symptoms than those from the lowest quintile (Hernández-Vásquez et al., 2020). Several underlying pathways could vary dependent on age. In China, Zou, Xu, Hong, and Yuan (2020) found that social support and optimism independently mediated this linkage in adolescents. In German adults, perceptions of social status partially mediated the association between objective SES and depressive symptoms, which may reflect financial stress (Hoebel et al., 2017). Conversely, in older adults in Finland, Poland, and Spain, material and behavioral factors were significant mediators of this association (Domènech-Abella et al., 2018). Studies in India and Iran found that the prevalence of MDD was higher in poor older adults compared with rich older adults (Azizabadi, Aminisani, & Emamian, 2022; Muhammad, Skariah, Kumar, & Srivastava, 2022). Interestingly, inequality was significantly higher among older people in Peru. In summary, people with low levels of SES were three times more likely to acquire a mental disorder (Safran et al., 2009), which implies that this phenomenon is not constrained to depressive symptoms.

We found significant inequalities in depressive symptoms according to age, marital status, and area and department of residence. Being older, previously married, living in Lima Metropolitan area or the Highlands (compared with the Coast or the Jungle), and, especially, residing in the south and southeast regions of the country appeared as relevant variables for pro-poor inequalities in depressive symptoms in Peru. These results point to the importance of the socioeconomic and spatial distribution of cases with depressive symptoms and access to treatment along with other demographic variables, which were significant and persistent across the period of analysis. In addition, socioeconomic inequality in access to treatment accross Peru coincides with that in other Latin American countries such as Brazil (Mrejen et al., 2022), Chile, Ecuador, and Colombia (Quijada, Villagrán, Vaccari Jiménez, Reyes, & Gallardo, 2018). Although 1 out of 10 Peruvians with depressive symptoms reported receiving depression treatment, the majority tended to belong to the wealthiest brackets. Despite the limited number of studies that evaluate this issue, this inequality could be attributed to a few structural problems that emerged from the Peruvian health care system. Out-of-pocket spending on health care is relatively low among the poorest households, which has led to their nearly exclusive use of the services provided by primary care providers (Pavone & Sánchez, 2018). Unfortunately, psychotropic medications, which are sold only under medical prescription, are frequently unavailable in primary care facilities and hospitals (Hodgkin, Piazza, Crisante, Gallo, & Fiestas, 2014). Moreover, the prices of psychotropic drugs are highly variable in the Peruvian setting (Valle, 2020). Another concerning aspect is that the referral system toward high-complexity facilities, where the majority of mental health professionals are concentrated, is full of bureaucracy and warrants long wait times. Given that these hurdles threaten accessibility to psychotropic medications, structural changes in the Peruvian health care system are required, especially after the COVID-19 pandemic, which left the healthcare system with structural, financial, and accessibility challenges. These challenges include the deepening of pro-poor inequalities in effective access to care, wait lists, and additional post-COVID-19 needs in the population.

The field of spatial analysis is still under constant development, and studies on inequalities in access to treatment for depression are few. The current results coincide with those published by other Peruvian studies that report persistently low proportion of treated cases in Cusco, Ayacucho, Puno, and Huancavelica (Villarreal-Zegarra et al., 2020). These departments also suffer from high vulnerability and poverty values but low educational attainment values. These patterns align with the issues discussed earlier, which strongly emphasize the role of SES and area of residence in depression. The current results also reveal the need for an in-depth understanding of the spatial dynamics of depressive symptoms at the national and periurban levels (Ruiz-Grosso et al., 2016). These findings highlight the relevance of the prioritization of financial and human resource needs for improving mental health, particularly with regard to territory.

### 4.3 Relevance to public health

Implementing multi- and inter-sectoral strategies is paramount to the reduction of disparities in access to treatment accross Peru. First, the country should consider the expansion of access to health care services. One strategy to overcome disparities in this regard is to increase the availability of specialized mental health care services. This aspect could be addressed, for example, by creating additional community centers or sheltered homes for mental health, hiring more mental health professionals, and implementing mobile health care programs to reach people in remote or hard-to-reach areas. Second, the country should ensure that specialized mental health care professionals are available in various regions in Peru. Currently, a few regions in Peru lack second-specialty mental health programs (García-Serna et al., 2022); thus, providing mental health training programs would ensure that trained human resources are available throughout the Peruvian health care system. Third, the social determinants of health, such as access to formal work, low levels of education, sex discrimination, unhealthy lifestyle, and violence, have been associated with access to treatment (Carod-Artal, 2017). Therefore, promoting social interventions for the improvement of the living conditions of the Peruvian population and generating social conditions for the reduction of inequality in mental health are necessary. Various social, political, and economic factors may influence disparities in access to treatment; thus, although reducing such inequalities is necessary for strengthening the health care system, it is insufficient. In addition, collecting further evidence of the reasons that underlie the lack of access to services for many people (particularly the poor) is urgently required. This area could be explored through qualitative or participatory research, as none has been published, thus far, in the country. The results of these studies could complement the current findings with rich experience-based evidence.

Apart from improving access to mental healthcare, a need also emerges to take further actions to directly target the social determinants of mental health out of which economic inequality appears to be a crucial one. In this sense, an opportunity exists to improve decision-making in terms of health policy that favors regions and departments that exhibited high levels of depressive symptoms based on SES. In the current analyses, the interconnection among place of residence, SES, and depressive symptoms was significant and persistent over time, which implies that a structural approach to health inequalities is urgently required. In addition, a better understanding of the factors that explain and the mechanisms that underlie the measurement of depressive symptoms is required to take effective public health actions on this matter, including the intersectionality of SES and mental health with various determinants such as gender, ethnicity, age, and migration status.

To properly interpret the results of the study in the context of decision-making on public health in Peru, understanding the Peruvian context is essential. The COVID-19 pandemic led to a reduction in the care capacity of the Peruvian mental health system, which prioritized cases related to COVID-19 (Villarreal-Zegarra et al., 2024). In turn, the prevalence of mental health problems increased during the pandemic, which disproportionately affected the most economically vulnerable populations (Villarreal-Zegarra, Reátegui-Rivera et al., 2023). However, the response of the Peruvian government for the mitigation of these mental health problems was inadequate, because the implementation of policies and technical guidelines was limited (Mayo-Puchoc et al., 2023). Consequently, the observed changes in inequality in the prevalence and access to treatment for mental health problems before and during COVID-19 may be analyzed given these factors.

### 4.4 Limitations and strengths

The results of the present study should be interpreted with a consideration of its limitations. First, although it measured depressive symptoms using a validated tool (Villarreal-Zegarra et al., 2019), doing so does not replace an assessment by a mental health professional; hence, misclassification may be an issue. Additionally, the estimates are representative of depressive symptoms that only presented in the two weeks prior to the survey. Second, the study was based on secondary data; thus, assessing other variables (e.g., risk behaviors, stigma, and type of treatment to improve mental health), which could be analyzed in future studies, was impossible. Third, in the absence of a direct measurement of SES (i.e., household income or consumption), we used the wealth index, which is a valid asset-based indicator used for measuring inequalities (McKenzie, 2005). The wealth index, which is strongly correlated with other asset-based measures (Mayfour & Hruschka, 2022), is a widely-used objective measure of SES in low- and middle-income countries. However, it is influenced by household- and community-level factors (Howe, Hargreaves, Ploubidis, De Stavola, & Huttly, 2011). Fourth, the Kulldorff spatial analysis technique only identifies circular-shaped clusters. Thus, certain low-risk areas could have been included in the clusters, while irregular-shaped clusters could have been overlooked (Tango, 2021). However, several spatial analysis techniques were used, including hotspot analysis (Getis-Ord-Gi∗) and the ordinary Kriging interpolation, which provided robustness to the findings. Fifth, notably, the analysis considered individuals who sought treatment in the past 12 months, while the assessment of depressive symptoms focused on the past two weeks. In other words, we did not consider individuals who (a) experienced depressive symptoms at certain points in the past year but may have recovered, (b) were unaware of their diagnosis, which prevented them from receiving appropriate care, or (c) presented with somatic-type depressive symptoms, which can be mistaken for physical health problems. Sixth, conducting a specific analysis at smaller administrative levels (i.e., districts, neighborhoods) was impossible as the Peruvian DHS is only representative at the national, regional and area of residence (urban, rural) levels. Seventh, although PHQ-9 has been validated in the Peruvian population for people with 16 years to more, we do not identify a study that validates the PHQ-9 in people with 15 years. If PHQ-9 may have different psychometrics properties in adolescents compared to adults, we considered it unlikely that there are different psychometric properties between adolescents of 16–17 years and 15 years. Therefore, we considered the PHQ-9 to be a valid scale for our participants. Eighth, this study used a cutoff of ≥10 points to identify cases of depressive symptoms. However, the results may have been different if the cutoff were modified to include only those with severe depressive symptoms (≥15 points). Although this option may be viable, sensitivity (14.3) and specificity (97.3) analyses in the Peruvian context for severe depressive symptoms render the PHQ-9 a very specific scale (Villarreal-Zegarra, Barrera-Begazo et al., 2023); as such, it would only estimate a small population, because this condition is less prevalent. Another possibility would be to consider depressive symptoms as a numerical variable, but doing so would make interpretation extremely difficult. Accordingly, the authors believe that using the cutoff of ≥10 points is the most conservative decision for spatial and inequality analyses.

Nonetheless, the present study also has its strengths. First, it used large sample sizes with national, regional and area of residence representativeness, which ensured sufficient statistical power. It also analyzed DHS data across 8 years and found consistent results, which rendered robustness to the findings by capturing variability and trends across years. Additionally, the screening tool for depressive symptoms has been previously validated for use in the Peruvian population and has demonstrated positive psychometric properties (Villarreal-Zegarra et al., 2019). The current study is one of the first in Latin America to assess depressive symptoms from the standpoints of socioeconomic inequality and spatial pattern.

## 5 Conclusion

Depressive symptoms were more concentrated among the poorest populations, whereas access to treatment was remarkably more concentrated among the wealthiest populations. Both patterns were steadily distributed between 2014 and 2021. The study observed a clustered spatial pattern in both years and identified similar high-risk areas. The predicted prevalence of depressive symptoms was heterogeneous within and between departments with several high-risk clusters identified in the southeastern areas of Peru. Hence, social policies that reduce socioeconomic and spatial inequalities in depressive symptoms and their treatment are urgently required.

## CRediT authorship contribution statement

**David Villarreal-Zegarra:** Writing – original draft, Visualization, Software, Investigation, Formal analysis, Data curation, Conceptualization. **Ali Al-kassab-Córdova:** Writing – review & editing, Writing – original draft, Visualization, Validation, Software, Methodology, Investigation, Formal analysis, Data curation, Conceptualization. **Sharlyn Otazú-Alfaro:** Writing – original draft, Investigation, Conceptualization. **Baltica Cabieses:** Writing – review & editing, Validation, Supervision, Methodology, Investigation, Conceptualization.

## Ethical statement

None of the patients or members of the general public were involved in the design, conduct, reporting or dissemination plans of our research. The Peruvian DHS database used in our study is free and accessible to the general public. The INEI was responsible for data collection, and this process did not represent an ethical risk for participants. INEI requested informed consent from participants 18 years old and above to obtain the information required in the survey. In the case of minors (17 years old and younger), the request for consent to evaluate the child was read to one of their parents or legal guardians.

## Funding source

This research did not receive any specific grant from funding agencies in the public, commercial, or not-for-profit sectors.

## Declaration of competing interest

None.

## Figures and Tables

**figs1:**
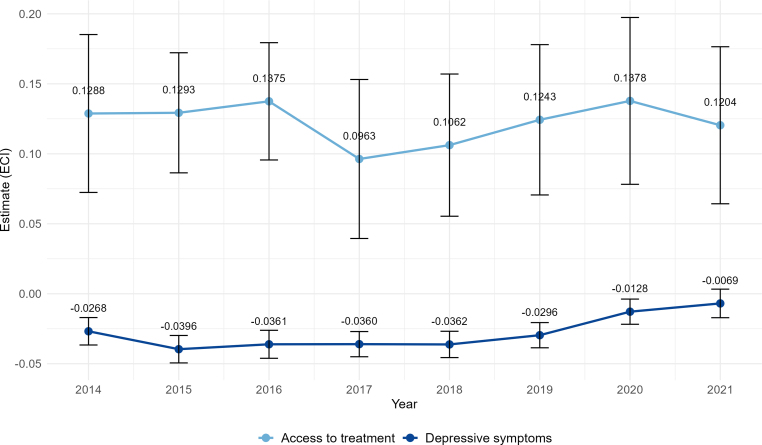
**Supplementary 2.** Trend of inequalities in depressive symptoms and its treatment in Peru from 2014 to 2021

**figs2:**
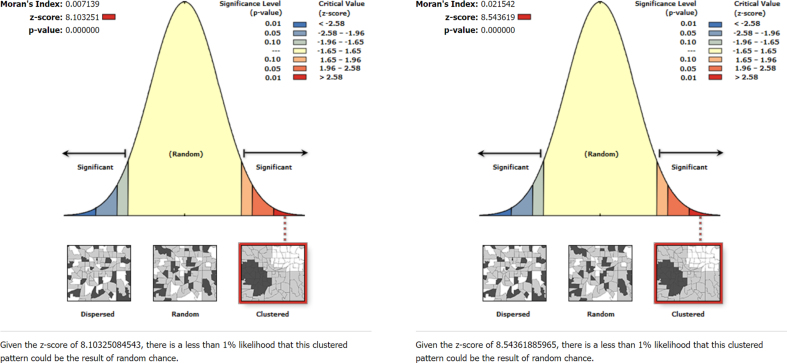
**Supplementary 3.** Global Moran’s I (spatial autocorrelation) of depressive symptoms in Peru in 2014 (left) and 2021 (right)

## Data Availability

Data are available in a public open-access repository. The database is accessible from the website of the National Institute of Statistics and Informatics (http://iinei.inei.gob.pe/microdatos/). The information can be obtained by entering the survey query tab and selecting the ENDES data using the health module data. Only cross-sectional information from 2014 to 2021 for the ENDES Health Questionnaire was used.
