# Supplementary material for: Socioeconomic and spatial distribution of depressive symptoms and access to treatment in Peru: A repeated nationwide cross-sectional study from 2014 to 2021
Source: SSM Popul Health. 2024 Nov 15;29:101724. doi: 10.1016/j.ssmph.2024.101724 (PMC11667185; doi:10.1016/j.ssmph.2024.101724)
Supplement: Multimedia component 1 [file mmc1.docx]

**Supplementary 1.** Comparison between the 2021 measurement and the expected population values in Peru

|  |  | 2021 | | National estimate by 2021 | |  |
| --- | --- | --- | --- | --- | --- | --- |
|  |  | (n=32,436) | | (n=24,928,336) | | p |
|  |  | n | % | n | % |  |
| Sex | Male | 13,863 | 48.3% | 12,289,306 | 49.3% | 0.887 |
|  | Female | 18,573 | 51.7% | 12,639,030 | 50.7% |  |
| Age group | 15-34 | 15,881 | 42.9% | 10,496,689 | 42.1% | 0.998 |
|  | 35-54 | 10,901 | 34.3% | 8,568,352 | 34.4% |  |
|  | 55-74 | 4,546 | 17.9% | 4,64,2053 | 18.62% |  |
|  | 75+ | 1,108 | 5.0% | 1,221,242 | 4.9% |  |

Note: p-value of Chi-Square test.
